# The human photosensitive epilepsy model for clinical proof‐of‐principle trials of novel antiseizure medications: 2. Analysis of drug trials and predictive value of the model

**DOI:** 10.1111/epi.18444

**Published:** 2025-05-10

**Authors:** Wolfgang Löscher, Dorothée Kasteleijn‐Nolst Trenité

**Affiliations:** ^1^ Translational Neuropharmacology Lab, NIFE, Department of Experimental Otology of the ENT Clinics Hannover Medical School Hannover Germany; ^2^ Department of Neurosurgery and Epilepsy University Medical Center Utrecht Utrecht the Netherlands; ^3^ NESMOS Department, Faculty of Medicine and Psychology Sapienza University Rome Italy

**Keywords:** animal models, intermittent photic stimulation, mechanism of action, pharmacodynamics, pharmacokinetics, photoparoxysmal EEG response, proof of concept

## Abstract

Clinical development of novel antiseizure medications (ASMs) would benefit from an early proof of principle (POP) model. The photosensitivity model, which uses the photoparoxysmal electroencephalographic response (PPR) as a surrogate of seizures, is currently the only human model that allows POP trials of investigational compounds after a single drug administration. Typically, trials in this model are performed as single‐blinded, placebo‐controlled phase IIa POP studies, evaluating a range of doses in small groups of epilepsy patients. In the second part of this review, based on the background information provided in Part 1, we analyze the outcome of all published trials performed over roughly 50 years. Many of the 35 drugs tested in the model were also examined in traditional add‐on trials in patients with epilepsy, thus allowing analysis of the predictivity of the model. Drugs were categorized into three groups: drugs that suppressed PPR; drugs that exerted no effect on PPR; and drugs that increased PPR, indicating a proconvulsant effect. For the vast majority of drugs, the model correctly predicted the drugs' activity during long‐term studies in patients with different types of epilepsy, including focal onset epilepsies. For some investigational compounds, the model detected proconvulsant activity that had not been observed in preclinical animal experiments and phase I studies in healthy volunteers, demonstrating the value of the model for adverse event assessment in patients with epilepsy. Limitations of the model are that it does not predict the extent of drug resistance of patients' seizures during chronic administration or efficacy differentiation of the novel drug from existing ASMs. Photosensitive POP trials are a useful tool to quantitatively predict drug efficacy and in aiding dose selection for subsequent larger phase IIb trials with chronic drug administration.

**Key points**

- PPRs are subtle ictal phenomena found in all types of epilepsy and at all ages.
- ASMs with diverse mechanisms of action are effective in suppressing PPRs.
- There is a highly significant correlation between effective doses to suppress PPRs and effective doses in the treatment of patients with epilepsy.
- The potency of ASMs to suppress PPRs is predicted by antiseizure potencies in the audiogenic seizure‐prone DBA/2 mouse.
- Drug testing in the photosensitive model is a useful tool to predict drug efficacy in both generalized and focal types of epilepsy.

## 1 INTRODUCTION

The human photosensitivity model is widely used for proof of principle (POP) trials on investigational antiseizure medications (ASMs).^1^, ^2^ In this model, photosensitive epilepsy patients are exposed to intermittent photic stimulation (IPS), which induces a photoparoxysmal electroencephalographic (EEG) response (PPR) as a surrogate of seizures. For testing of ASMs, the partial or complete abolishment of PPR is used as an endpoint. As described in the first part of this review, the model was developed some 50 years ago and since then has been standardized and validated by numerous clinically used ASMs.^3^

In the second part of this review, we evaluate the clinical studies on approved and investigational ASMs that have been performed with the human photosensitivity model. We will show that IPS testing of photoparoxysmal response in patients with epilepsy provides a useful clinical “model” for POP phase IIa trials, allowing rapid translation of demonstrated preclinical antiseizure activity to patients and prediction of doses that may be effective in chronic phase IIb and phase III clinical trials in patients with drug‐resistant focal or other types of epilepsy. Importantly, the data include examples of novel drugs with antiseizure activity in animal models failing in POP phase IIa trials in the photosensitivity model in epilepsy patients and subsequently failing in phase IIb trials. Therefore, we included a section that discusses which animal models best predict subsequent drug effects in the human photosensitivity model and may help in dose selection for clinical trials.

## 2 PUBLISHED DATA ON THE EFFICACY OF ASMs IN THE PHOTOSENSITIVITY MODEL

Table 1 summarizes the outcome of trials with 13 approved ASMs in the photosensitivity model in epilepsy patients. For six ASMs (progabide, vigabatrin, lamotrigine, levetiracetam [LEV], brivaracetam [BRV], cenobamate), the trial in photosensitive epilepsy patients was either the first POP trial or among the first clinical trials with these drugs long before approval in either the United States or Europe. For all six ASMs, the positive data in the photosensitivity model correctly predicted their clinical antiseizure efficacy.

**TABLE 1:** Effect of approved antiseizure medications in the photosensitivity model versus the antiseizure effect of these drugs in patients with different types of epilepsy.

ASM	Company/sponsor	Main MOA	Photosensitivity model in epilepsy patients				Antiseizure effect in epilepsy patients		First approval in the USA or Europe	References for photosensitivity model
			Trial/publication	Patients, *N* (M/F)	Oral doses, mg	Result, (PPR reduction/abolition)	Focal onset	IGE/DEE		
Valproate	Sanofi‐Synthélabo	Multiple	1978/1979 + 1986	18 (3/15)	600, 900	+	+	+	1967	4
Valproate	−	Multiple	?/1987	6 (5/1)	1000	+	+	+	1967	5
Valproate	Abbott Labs	Multiple	2008/2008 (A)	10	5 mg/kg iv initially, then 1.145 mg/kg/h for 12 h	+	+	+	1967	6
Valpromide^a^	SEIN	Multiple	1980/1986	2 (0/2)	900	+	+	+	1967	4
Diazepam	SEIN	GABAAR PAM	1979/1986	7 (4/3)	5–10 iv	+	+	+	1963	4
Ethosuximide	SEIN	T‐type Ca^2+^ channel ⇩	?/1986	4 (3/1)	400 (fluid)	+	−	+	1958	4
Primidone^b^	SEIN	GABAAR PAM	?/1986	3 (0/3)	500	+	+	+	1954	4
Carbamazepine	SEIN	I_NAT_ ⇩	?/1986	4 (2/2)	400 (fluid)	+	+	−/+	1964	4
Carbamazepine	Epilepsy Project	I_NAT_ ⇩	2011/2014	6 (3/3)	400 (tablets)	−	+	−/+	1964	7
Mephenytoin (methoin)	SEIN	I_NAT_ ⇩	?/1986	4 (2/2)	400	+	+	+	1947	4
Progabide^c^	LERS‐Synthélabo	GABAAR PAM	1981/1986	13 (4/9)	600–2700	+	+	+	1985	4
Vigabatrin	Merrell Dow	GABA‐T inhibitor	1986/1987	6 (1/5)	3000	+	+	+	1989	5
Lamotrigine	Burroughs Wellcome	I_NAT_ ⇩	1985/1986	6 (4/2)	120, 240	+	+	+	1990	4
Levetiracetam	UCB Pharma	SV2A modulator	1995/1996	12 (2/10)	250–1000	+	+	+	2000	8
Levetiracetam	Epilepsy Project	SV2A modulator	2011/2014	6 (3/3)	1000	+	+	+	2000	7
Levetiracetam	UCB Pharma	SV2A modulator	2018/2020	9 (3/6)	1500 iv	+	+	+	2000	9
Brivaracetam	UCB Pharma	SV2A modulator	2004/2007	19 (4/15)	10–80	+	+	+	2016	10
Brivaracetam	UCB Pharma	SV2A modulator	2018/2020	9 (3/6)	100 iv	+	+	+	2016	9
Cenobamate	SK Life Science	I_NAP_ ⇩ + GABAAR PAM	2008/2019	7 (2/5)	100–400	+	+	+	2019	11

*Note*: See Löscher and Klein^12^ for antiseizure effects in epilepsy patients. In the PPR column, reduction or abolition of PPR is indicated by “+” and lack of such effect by “−”. Dates of first approval in the USA or Europe are from Löscher et al.^13^

Abbreviations: A, abstract; ASM, antiseizure medication; DEE, developmental and epileptic encephalopathy; F, female; GABAAR, γ‐aminobutyric acid type A receptor; GABA‐T, γ‐aminobutyric acid aminotransferase; IGE, idiopathic generalized epilepsy; I_NAP_, persistent sodium current; I_NAT_, transient sodium current; iv, intravenous; M, male; MOA, mechanism of action; PAM, positive allosteric modulator; PPR, photoparoxysmal electroencephalographic response; SEIN, Stichting Epilepsie Instellingen Nederland; SV2A, synaptic vesicle glycoprotein 2A.

^a^ Approved for epilepsy in Italy.

^b^ Metabolized to phenobarbital.

^c^ Approved for epilepsy in France.

The synaptic vesicle glycoprotein 2A (SV2A) modulators LEV and BRV are important examples of the predictive value of POP trials in the photosensitivity model. During the preclinical development of LEV in the late 1980s and early 1990s, this compound was found to be inactive in the gatekeeper animal models of the time, namely, the maximal electroshock seizure (MES) test and the subcutaneous pentylenetetrazole (PTZ) seizure test.^14^ This lack of preclinical antiseizure efficacy in standard models was confirmed by the National Institute of Neurological Disorders and Stroke‐funded Anticonvulsant Screening Program (ASP), and the program director did not recommend further development of LEV.^15^ On the other hand, LEV was quite effective in a genetic model of generalized epilepsy, the audiogenic seizure‐prone mouse,^16^ and in the amygdala kindling model of focal onset seizures.^14^ These findings revealed a unique profile that made LEV the first known ASM to display efficacy in fully kindled rats while showing the lack of antiseizure activity in the two major preclinical screening models (MES, PTZ) used for decades to identify new ASM candidates. Thus, because of this novel preclinical profile, LEV was termed an “atypical antiepileptic drug” (Klitgaard, 2001)^17^. Based on these data, Löscher and Hönack^14^ proposed that LEV should be particularly effective against focal seizures. Subsequent experiments by the same group indicated that LEV may also exert disease‐modifying effects.^18^ These findings significantly contributed to the decision to start clinical trials with LEV. In 1995, the first phase IIa POP trial with LEV was conducted in photosensitive patients,^8^ and this trial correctly predicted the clinical efficacy of LEV seen in subsequent phase IIb and pivotal phase III add‐on trials in patients with drug‐resistant focal onset epilepsy.^19^ Similarly, the first POP trial with BRV, a more potent analog of LEV, was performed in the photosensitivity model^10^ and correctly predicted the antiseizure effect of this drug in subsequent pivotal clinical trials.^20^, ^21^

For the other seven ASMs of Table 1, clinical efficacy in photosensitive epilepsy patients was evaluated to validate the predictivity of the model. As shown in Table 1, the positive response of these seven ASMs in the photosensitivity model was in line with their clinical efficacy against focal and/or generalized seizures. Importantly, ASMs such as ethosuximide (ESM) that act primarily against generalized seizures were effective in the photosensitivity model, as were ASMs that act primarily against focal onset seizures. Thus, the model predicts antiseizure efficacy against different types of seizures and epilepsy. Furthermore, ASMs with diverse mechanisms of action (MOAs) were effective in the model, indicating that the model is not particularly sensitive to a specific MOA (but see Section 4). However, the PPR does not predict against which type(s) of epilepsy (focal vs. generalized or both) the novel drug will be effective, or whether the drug will have a narrow (e.g., ESM) or broad spectrum of efficacy.

For some of the ASMs listed in Table 1, trials in the photosensitivity model were repeated, for instance, to compare different routes of administration (oral vs. intravenous). Furthermore, in some trials, different ASMs were compared, for example, in a trial comparing the onset of effect of intravenous LEV versus intravenous BRV^9^ or evaluating a narrow‐spectrum (carbamazepine [CBZ]) versus a broad‐spectrum (LEV) ASM.^7^ In the latter trial, there was no evidence of significant PPR suppression with CBZ, which contrasts with the marked suppressive CBZ effect reported earlier by Binnie et al.^4^ with the same single oral dose of 400 mg. A possible explanation for the difference between the two studies is that Binnie et al.^4^ administered CBZ as a liquid for rapid absorption, whereas French et al.^7^ used overencapsulated CBZ tablets, resulting in retarded absorption and low CBZ plasma levels. Thus, if patients were given CBZ as a liquid, or IPS testing would not have stopped at 6 h postdose (as occurred in the French et al.^7^ study) instead of the usual testing over 32 h in the photosensitivity model, the trial outcome may have been different.^22^, ^23^

As described in Part 1 of the review, not all photosensitive epilepsy patients show a PPR suppressant effect with a given ASM, as occurs with drug‐resistant epilepsy in clinical practice. Table 2 summarizes the acute responses to the ASMs shown in Table 1. The percentage of photosensitive patients not responding with PPR suppression to ASMs ranges from 0% to 83% (median = 0). At least in part, this large range in the PPR response to ASMs is a result of trial design that aims to find the lowest effective dose. Nevertheless, the data in Table 2 indicate that PPR suppression response and failure to suppress PPR with a given ASM do not predict clinical resistance observed with these drugs.^24^, ^25^, ^26^, ^27^ This limitation of the photosensitivity model will be discussed in Section 8.

**TABLE 2:** Effects of approved antiseizure medications on PPR in epilepsy patients.

Drug	Dose, oral	Experiments	Response			Percent no response	Reference
			PPR reduced	PPR abolished	No response		
Diazepam	5–10 (iv)	7	0	7	0	0	4
Valproate	600	8	2	3	3	38	4
	900	10	2	4	4	40	
Valproate	1000	6	3		3	50	5
Valpromide	900	2	1	1	0	0	4
Ethosuximide	400	4	1	3	0	0	4
Primidone	500	3	0	2	1	33	4
Mephenytoin	400	4	0	2	2	50	4
Progabide	1200–2700	7	0	4	3	43	4
Carbamazepine	400	4	2	2	0	0	4
Carbamazepine	400	6	1	–	5	83^a^	7
Vigabatrin	3000	6	3		3	50	5
Lamotrigine	120	1	1	0	0	0	4
	240	5	3	2	0	0	
Levetiracetam	250	4	3	0	1	25	8
	500	2	1	0	1	50	
	750	2	0	2	0	0	
	1000	5	1	4	0	0	
Levetiracetam	1000	5	2	3	0	0	7
Brivaracetam	10	4	1	3	0	0	10
	20	5	1	4	0	0	
	40	5	2	3	0	0	
	80	5	0	5	0	0	
Cenobamate	100	3	1	0	2	67	11
	250	4	3	1	0	0	
	400	4	1	1	2	50	

*Note*: See Table 1 for further details. Abbreviations: iv, intravenous; PPR, photoparoxysmal electroencephalographic response.

^a^ Retarded absorption of carbamazepine and intermittent photic stimulation testing stopped at 6 h postdose (instead of the usual testing over 32 h in this model) are likely explanations of the negative outcome (see text).

Various other ASMs that were approved in recent decades were either not evaluated in the photosensitivity model or respective trials were planned but not finished. For instance, attempts have been made to have a PPR trial with topiramate (Janssen‐Cilag), tiagabine (Novo Nordisk), zonisamide (Eisai), lacosamide (Schwarz/UCB), pregabalin (Pfizer), and oxcarbazepine (Novartis).

## 3 CORRELATION BETWEEN ACUTE DOSES OF ASMs IN THE PHOTOSENSITIVITY MODEL AND CHRONIC MAINTENANCE DOSES IN EPILEPSY PATIENTS

**FIGURE 1:**
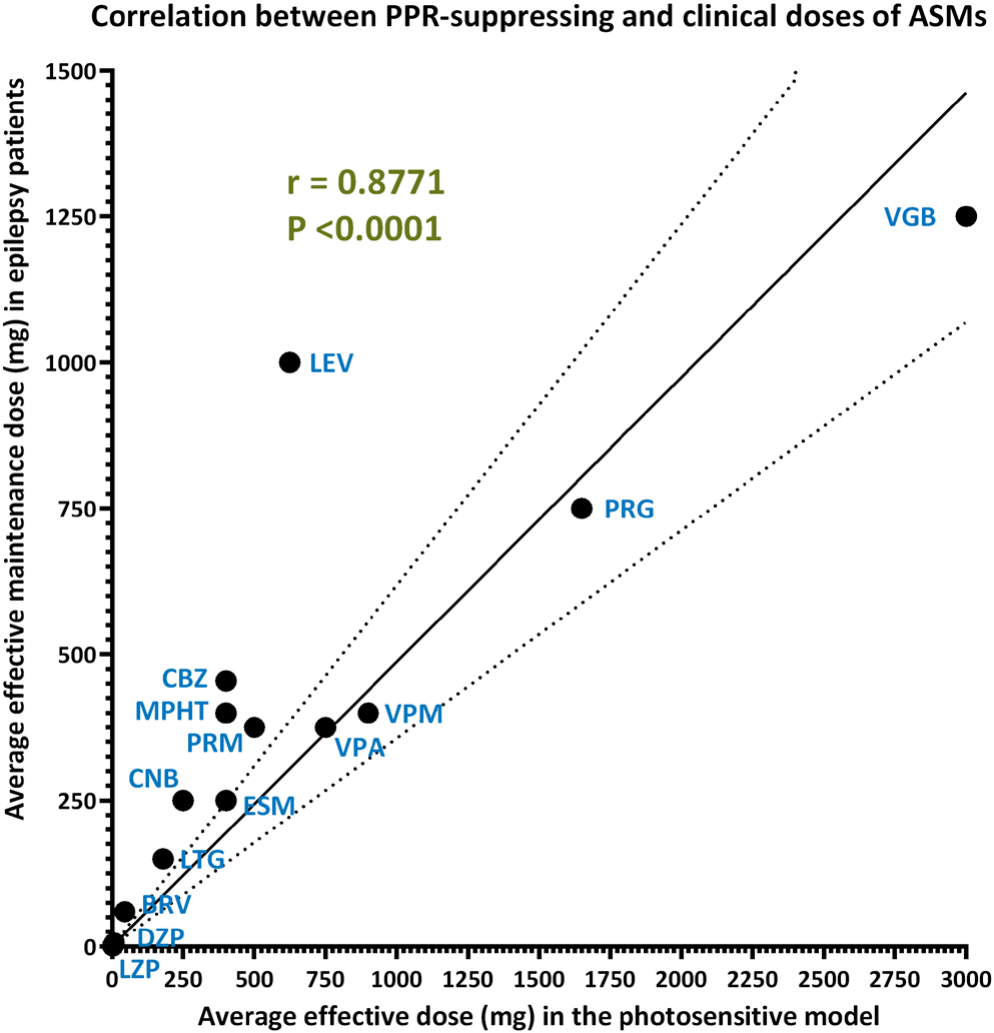
Correlation between the average effective single doses of 14 approved antiseizure medications (ASMs) that suppress photoparoxysmal electroencephalographic responses (PPRs) in photosensitive epilepsy patients and the average effective maintenance dose administered during chronic treatment of patients with epilepsy. Note that during maintenance treatment, these doses are typically administered twice or three times per day, depending on the pharmacokinetics of the respective drug. For six of the ASMs shown, the efficacy in the photosensitivity model was determined long before clinical maintenance doses were established in patients with epilepsy. See Table 3 for the doses that were used for the correlation analysis. Spearman rank correlation was used to calculate the correlation coefficient *r*. Linear regression analysis was used to construct the solid line through the data and the standard errors as indicated by the stippled lines. BRV, brivaracetam; CBZ, carbamazepine; CNB, cenobamate; DZP, diazepam; ESM, ethosuximide; LEV, levetiracetam; LTG, lamotrigine; LZP, lorazepam; MPHT, mephenytoin; PRG, progabide; PRM, primidone; VGB, vigabatrin; VPA, valproate; VPM, valpromide.

Achieving complete seizure control is the most important objective in the treatment of epilepsy. For this goal, ASMs are administered chronically to prevent seizure recurrence in patients with spontaneous recurrent seizures.^12^ Depending on the pharmacokinetics (PK) of the respective ASM, it is given once, twice, or three times daily. Table 3 shows the daily oral doses and average single doses of 14 ASMs used in the treatment of epilepsy. For comparison, the effective oral doses in the photosensitivity model are shown. We used these data to analyze whether the average acute single doses shown to suppress PPR in the photosensitivity model would predict the average doses of ASMs used in the treatment of epilepsy. As shown in Figure 1, a highly significant correlation was obtained, with a Spearman *r* of .8771 (95% confidence interval = .6378–.9619, *p* < .0001). This finding suggests that testing of investigational ASMs in the photosensitivity model not only predicts antiseizure efficacy in subsequent phase II and III clinical trials in patients with epilepsy but also provides important information for dose selection for these trials.

**TABLE 3:** Effective doses of approved ASMs in the photosensitivity model and in the chronic treatment of patients with epilepsy.

ASM	Single oral dose effective in the photosensitive model in adult epilepsy patients, mg		Daily maintenance dose for antiseizure effect in adult epilepsy patients	
	Range of doses tested	Average acute dose^a^	Common range of doses and number of applications per day	Average single dose (given 1–3 times daily)^a^
Brivaracetam	10–80	45	25–100 b.i.d.	60
Carbamazepine	400	400	300–600 t.i.d.	455
Cenobamate	100–400	250	100–400 q.d.	250
Diazepam	5–10 (iv)	7.5	2–10 b.i.d.	6
Ethosuximide	400	400	250 b.i.d./t.i.d.	250
Lamotrigine	120, 240	180	100–200 b.i.d.	150
Levetiracetam	250–1000	625	500–1500 b.i.d.	1000
Lorazepam^b^	2	2	.5–2 (rescue medication)	1.25
Mephenytoin (methoin)	400	400	200–600 q.d.	400
Primidone	500	500	250–500 t.i.d.	375
Progabide^c^	600–2700	1650	500–1000 t.i.d.	750
Valproate	600, 900	750	250–500 t.i.d.	375
Valpromide^d^	900	900	300–500 t.i.d.	400
Vigabatrin	3000	3000	1000–1500 b.i.d.	1250

*Note*: See Table 2 for the effects of these drugs in the photosensitivity model and respective references. Common daily maintenance doses in patients with epilepsy are from Horváth et al.^28^ and Vossler et al.^29^ See Figure 1 for correlation analysis. Abbreviations: ASM, antiseizure medication; b.i.d., twice daily; iv, intravenous; q.d., once daily; t.i.d., three times daily.

^a^ Mean of the dose range.

^b^ Approved for status epilepticus.

^c^ Approved for epilepsy in France.

^d^ Approved for epilepsy in Italy.

## 4 PUBLISHED DATA ON THE EFFICACY OF INVESTIGATIONAL DRUGS IN THE PHOTOSENSITIVITY MODEL

Table 4 summarizes the outcome of phase IIa trials with 17 investigational drugs in the photosensitivity model in epilepsy patients. Some of these drugs were subsequently evaluated in phase IIb add‐on trials, thus allowing examination of whether the photosensitivity model provided predictive data. Furthermore, several other drugs (methohexital, secobarbital, flumazenil, lorazepam, alprazolam), which are approved for other indications than epilepsy but were tested in the photosensitivity model, are shown in Table 4.

**TABLE 4:** Effect of investigational compounds and drugs approved for other indications in the photosensitivity model.

Drug	Company/sponsor	Main MOA	Preclinical potency against audiogenic seizures in DBA/2 mice, (ED_50_ in mg/kg ip; clonus)	Photosensitive model in epilepsy patients				Confirmation of antiseizure effect in epilepsy patients		References for the clinical studies
				Trial/publication	Patients, *n* (M/F)	Oral doses, mg	Result (PPR reduction/abolition)	Focal onset	IGE/DEE	
Nafimidone	SEIN	Na^+^ channel modulation and cholinesterase inhibition?	ND	?/1986	4 (4/0)	200, 400	−	+ (but increases CBZ and PHT levels)	ND	4, 30
R57720	Janssen/SEIN	ND	ND	1985/1986	3 (1/2)	80, 160	+	ND	ND	4
Methohexital^a^	SEIN	Multiple	ND	?/1986	2 (1/1)	50 (iv)	−	ND	ND	4
Secobarbital^a^ (quinalbarbitone)	SEIN	GABAAR PAM	ND	?/1986	14 (4/10)	200 (iv)	−	ND	ND	4
Taltrimide	Leiras‐Medica	Taurine derivative	ND	1986/1987	8	1000–2000 for 6 days	PPR⇧	Proconvulsant	Proconvulsant	31, 32
Loreclezole	Janssen	GABAAR PAM	22.3	1988/90	5 (2/3)	100–150	+	+	ND	33, 34, 35
ZK 95962	Schering/Bayer	GABAAR PAM	.2	1989/90	6 (3/3)	20–40 μg/kg iv	+	ND	ND	36
Org 6370	Organon	ND	ND	1988/92	4	50–500	PPR⇧	Proconvulsant in the PPR trial^b^	Proconvulsant in the PPR trial^b^	37
Flumazenil	Hoffmann‐LaRoche	GABAAR PAM^c^	6	1993/2016	8 (4/4)	30–100	+	+	+	38, 39, 40
GABA + PS^d^	University of Genoa + Fidia	Augmentation of GABAergic transmission?	ND	1993/94	9 (3/6)	3000 (GABA) + 600 or 1200 (PS)	−	−	+ (absences only)	41, 42
Abecarnil	Schering/Bayer	GABAAR PAM	.0034 (tonic)	1995/2016	4 (3/1)	5–10	+	ND	ND	40
Carisbamate	J&J/Janssen	Multiple	ND	2003/07	18 (14/4)	500–1000	+	+/− (phase III)	Phase II ongoing	43, 44, 45, 46
Pitolisant^e^	Bioproject	Histamine H3 receptor antagonist	ND	2004/13	14 (3/11)	20–60	+	−	ND	47, 48
Seletracetam	UCB Pharma	SV2A modulator	.17	2006/25	27 (5/22)	.5–20	+	+	ND	49, 50
JZP‐4^f^	Jazz Pharmaceuticals	Ca^2+^ and Na^+^ channel blocker	ND	2008/A	4 (3/1)	25–200	+	ND	ND	51
JNJ‐26489112	J&J	Multiple	21	2008/14	12 (3/9)	1000‐3000	+	ND	ND	52
ICA‐105665	ICAgen	Kv7.2–7.5 activator	.91	2010/13	13 (4/9)	100–600	+	ND	ND	53
Selurampanel	Novartis	AMPA/kainate receptor antagonist	?^g^	2011/15	10 (2/8)	15–100	+	+	ND	54, 55
Darigabat	Pfizer/Cerevel Therapeutics	GABAAR PAM	ND	2017/19	7 (2/5)	17.5, 52.5	+	Phase II ongoing	ND	56
Lorazepam^h^	Bausch Health	GABAAR PAM	.002	2017/19	7 (2/5)	2	+	+ (seizure rescue therapy)	+ (seizure rescue therapy)	56, 57
Alprazolam^i^	UCB Pharma	GABAAR PAM	.08 (po)	?/2019	5 (0/5)	.5–2 (inhaled)	+	+ (inhaled)	+ (inhaled)	58, 59
Prax‐628	Praxis	I_NAP_ ⇩	ND	2023/24^j^	8	15, 45	+	ND	ND	Praxis, 2024^j^

*Note*: In the PPR column, reduction or abolition of PPR is indicated by “+” and lack of such effect by “−”. If known, the antiseizure effect of these drugs in patients with different types of epilepsy is shown for comparison, using again “+” or “−” to indicate efficacy or lack of efficacy, respectively. Furthermore, if known, antiseizure ED_50_s in audiogenic seizure‐prone DBA/2 mice are shown (see Section 5 and Appendix S1 for discussion).

Abbreviations: A, abstract; AMPA, α‐amino‐3‐hydroxy‐5‐methyl‐4‐isoxazolepropionic acid; CBZ, carbamazepine; DEE, developmental and epileptic encephalopathy; ED_50_, median effective dose; F, female; GABA, γ‐aminobutyric acid; GABAAR, GABA_A_ receptor; IGE, idiopathic generalized epilepsy; I_NAP_, persistent sodium current; ip, intraperitoneal; iv, intravenous; M, male; MOA, mechanism of action; ND, not determined (or not found in the public domain); PAM, positive allosteric modulator; PHT, phenytoin; po, oral; PPR, photoparoxysmal electroencephalographic response; PS, phosphatidylserine; SEIN, Stichting Epilepsie Instellingen Nederland; SV2A, synaptic vesicle glycoprotein 2A.

^a^ Sedative–hypnotic barbiturates were used to show that PPR cannot be suppressed by sedation.

^b^ Org 6370 induced epileptic seizures in the photosensitive patients.

^c^ Approved benzodiazepine antagonist with low partial agonist efficacy at GABAAR.

^d^ A liposomal suspension of PS and GABA (liposome‐entrapped GABA) has been shown to cross the blood–brain barrier and exert anticonvulsant effects in animal models.

^e^ Approved for treatment of narcolepsy.

^f^ Structurally related to lamotrigine.

^g^ Effective but data not in the public domain.

^h^ Approved for anxiety, status epilepticus, and premedication of anesthesia.

^i^ Alprazolam is approved for treatment of anxiety and panic disorders; alprazolam administered via the Staccato breath‐actuated device is under development for treating prolonged seizures or seizure clusters.

^j^ Press release by Praxis (https://investors.praxismedicines.com/news‐releases/news‐release‐details/praxis‐precision‐medicines‐reports‐positive‐results‐prax‐628/).

The 22 drugs listed in Table 4 can be subdivided into three groups: group 1, drugs (*n* = 16) that suppressed PPR; group 2, drugs (*n* = 4) that exerted no effect on PPR; and group 3, drugs (*n* = 2) that exacerbated PPR, indicating a proconvulsant effect. It is important to note that all drugs had antiseizure effects in animal models before the IPS POP testing in photosensitive patients.

The 16 compounds of group 1 include drugs that act by various MOAs (Table 4). The largest group (*n* = 8) are positive allosteric modulators (PAMs) of γ‐aminobutyric acid type A (GABA_A_) receptors, including the benzodiazepine (BDZ) antagonist flumazenil, which exerts low partial agonist efficacy at the GABA_A_ receptor^60^, ^61^ and has been reported to exert antiseizure effects in patients with epilepsy.^38^, ^39^ In line with this clinical finding, flumazenil partially suppressed PPR in photosensitive patients but was less effective than the GABA_A_ receptor PAM abecarnil,^40^ which has a higher intrinsic efficacy at the GABA_A_ receptor than flumazenil and acts as a full agonist at some, and a partial agonist at other, GABA_A_ receptor subtypes.^62^ As expected from these profiles, sedative adverse effects were observed after abecarnil but not flumazenil in the study in photosensitive patients.^40^

The subtype‐selective GABA_A_ receptor PAM darigabat is a promising new ASM, because this compound is selective for receptors containing α2/3/5 subunits compared with receptors containing the α1 subunit, which is thought to mediate sedative adverse effects of BDZs.^63^ In photosensitive patients, darigabat produced a marked reduction in PPR compared to the placebo that was similar in degree to the nonselective GABA_A_ receptor PAM lorazepam.^56^ Dizziness and somnolence were the commonest adverse events but were not severe.

For most compounds of group 1 that were also tested for chronic antiseizure effects, the positive response in the photosensitivity model was in line with their antiseizure efficacy in clinical trials. There was only one apparent exception, namely, pitolisant. This histamine H3 receptor antagonist, which is approved for the treatment of narcolepsy and excessive daytime sleepiness, suppressed PPR in the photosensitivity model^47^ but in a subsequent exploratory phase II add‐on clinical trial, performed to evaluate the antiseizure effect and safety of pitolisant in patients with drug‐resistant focal onset seizures, only one third of patients achieved a clinical response after 3 months of treatment, and thus pitolisant was considered insufficiently effective for further development.^48^ The authors concluded that no firm conclusions about the antiseizure efficacy of pitolisant can be drawn, because the number of included subjects (*n* = 23) was small and the study was not placebo‐controlled.

Carisbamate, an alkyl‐carbamate that acts by multiple MOAs, including GABAergic effects, antagonistic effects on glutamate receptor subtypes (α‐amino‐3‐hydroxy‐5‐methyl‐4‐isoxazolepropionic acid and [AMPA] N‐methyl‐D‐aspartate [NMDA]) receptors), and modulatory effects on voltage‐dependent sodium and calcium channels,^64^ suppressed PPR in photosensitive epilepsy patients.^43^ In a subsequent phase II study,^44^ carisbamate was effective as adjunctive therapy for reducing the frequency of focal onset seizures, thus confirming the prediction of the photosensitivity model. However, of two pivotal phase III add‐on studies in patients with drug‐resistant focal epilepsy, carisbamate was effective in only one.^45^ A third phase III study with higher doses did not demonstrate efficacy,^46^ and the development of focal onset seizures was halted. Carisbamate is currently being evaluated in patients with Lennox–Gastaut syndrome.^65^ Thus, it remains to be established whether the positive prediction of the photosensitivity model holds true for this drug.

**FIGURE 2:**
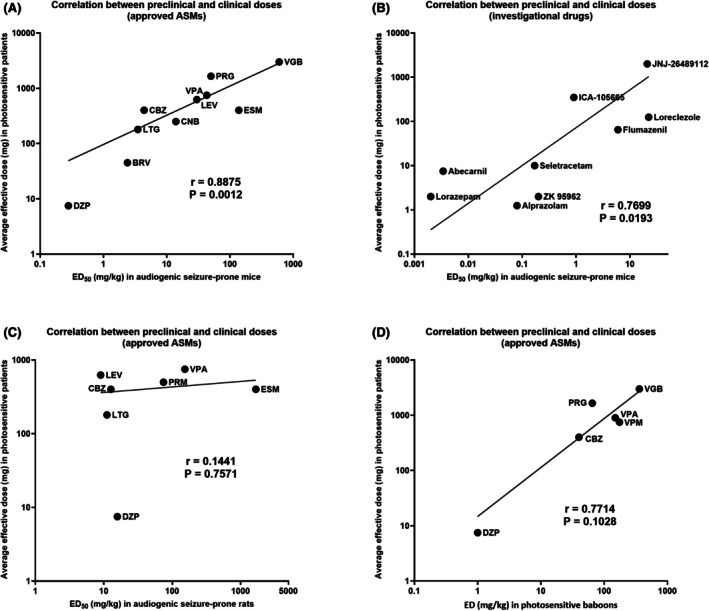
Correlation between preclinical antiseizure doses of drugs in animal models and the average effective dose to suppress the photoparoxysmal electroencephalographic response (PPR) in photosensitive epilepsy patients. Preclinical doses are given as median effective dose (ED_50_; or ED in baboons) in mg/kg intraperitoneally (or intravenously in baboons), whereas clinical doses are in mg orally (except for diazepam, which was administered intravenously). Spearman rank correlation was used to calculate the correlation coefficient *r*. Nonlinear regression analysis was used to construct the lines through the data. (A) Approved antiseizure medications (ASMs): correlation between anticonvulsant ED_50_s for suppression of clonic audiogenic seizures in the DBA/2 mouse model of reflex epilepsy and the average dose of ASMs suppressing PPR in patients with photosensitive epilepsy. Note that doses at both axes are shown on a log scale. The relative drug potencies were significantly correlated. (B) Investigational drugs: correlation between anticonvulsant ED_50_s for suppression of clonic audiogenic seizures in the DBA/2 mouse model of reflex epilepsy and the average dose of ASMs suppressing PPR in patients with photosensitive epilepsy. Note that doses at both axes are shown on a log scale. The relative drug potencies were significantly correlated. (C) ASMs: lack of significant correlation between anticonvulsant ED_50_s for suppression of clonic audiogenic seizures in the audiogenic seizure‐prone rat model of reflex epilepsy and the average dose of ASMs suppressing PPR in patients with photosensitive epilepsy. (D) ASMs: correlation between anticonvulsant EDs for suppression of photosensitive seizures in the epileptic baboon model of reflex epilepsy and the average dose of ASMs suppressing PPR in patients with photosensitive epilepsy. Note that two ASMs (ethosuximide and primidone) that were effective in photosensitive patients were ineffective in baboons and are therefore not included in the graph; thus, the correlation shown in panel D is false positive. See Tables 3, 4, 5 for individual values. BRV, brivaracetam; CBZ, carbamazepine; CNB, cenobamate; DZP, diazepam; ESM, ethosuximide; LEV, levetiracetam; LTG, lamotrigine; PRG, progabide; PRM, primidone; VGB, vigabatrin; VPA, valproate; VPM, valpromide.

The LEV analog seletracetam (SEL) is the most potent compound of group 1 and the most potent drug ever tested in the photosensitivity model, exceeding even BDZs such as diazepam, lorazepam, and alprazolam (Table 4, Figure 2). SEL abolished PPR at .5 mg (the lowest dose tested) with a rapid onset of effect^50^ due to its high lipophilicity and high affinity for SV2A.^15^ As suggested by Kasteleijn‐Nolst Trenité et al.,^50^ SEL may thus be suited as a seizure rescue medication with transmucosal (intranasal or buccal) administration without the inherent adverse effects of the BDZs (marked sedation, respiratory depression, abuse liability) that are currently used for this purpose. The promising effect of SEL continued in subsequent chronic phase IIa trials in patients with drug‐resistant focal onset seizures (Table 4).

Group 2 includes four drugs (methohexital, secobarbital, nafimidone, and a combination of GABA and phosphatidylserine [PS]) that exerted no effect on PPR (Table 4). Binnie et al.^4^ used the two sedative–hypnotic barbiturates secobarbital and methohexital, which are not used as ASMs, to demonstrate that marked sedation or hypnosis alone produces no reduction in photosensitivity, even where it was necessary to awaken the patient before performing IPS.

In contrast to GABA, which only poorly penetrates into the brain after systemic administration,^66^ a liposomal suspension of PS and GABA (GABA‐PS; liposome‐entrapped GABA) has been shown to cross the blood–brain barrier (BBB) and exert anticonvulsant effects in animal models such as isoniazid‐ and penicillin‐induced seizures in rats.^67^, ^68^, ^69^, ^70^ However, in the photosensitivity model, no suppressive effect on PPR was observed.^42^ Loeb et al.^41^ investigated the effect of GABA‐PS in a pilot add‐on study in 42 patients with drug‐resistant epilepsy. The group included patients with focal onset seizures and absence seizures. Patients with focal onset seizures showed no significant improvement; on the other hand, there was a remarkable decrease in absence seizures, linearly related to the dose of GABA‐PS. However, the two clinical pilot studies with GABA‐PS were not placebo‐controlled, which limits the interpretation of the apparent dichotomy in the effect of GABA‐PS in the two studies.^41^, ^42^

Another apparent dichotomy relates to the effect of nafimidone, which did not suppress PPR in photosensitive patients^4^ but exerted an antiseizure effect in a small clinical add‐on trial in 12 patients with drug‐resistant focal onset seizures.^30^ However, in the latter trial, nafimidone had a marked inhibitory effect on the clearance of CBZ and phenytoin (PHT), resulting in higher plasma levels of these ASMs and possibly the apparent efficacy of nafimidone.

Group 3 comprises two drugs (taltrimide and Org 6370) that increased PPR, indicating a proconvulsant effect. Because proconvulsant drug activity is considered a serious adverse effect, associated with significant safety concerns,^71^ these drugs will be discussed in detail in Section 6.

Overall, the data in Table 4 indicate that the MOA of a drug can lead to contrasting effects in the photosensitivity model, namely, no effect on PPR, suppression of PPR, or even an increase of PPR. Can such effects in the photosensitivity model, which is often the first POP trial in epilepsy patients, be predicted by any preclinical animal model? This important question will be discussed in the next section.

## 5 CORRELATION BETWEEN POP DATA IN THE HUMAN PHOTOSENSITIVITY MODEL AND PRECLINICAL DATA

Table 5 summarizes the antiseizure potencies of 15 first‐, second‐, and third‐generation ASMs in several seizure models, which are described in detail in Appendix S1. For comparison, the efficacy of these ASMs in the human photosensitivity model and in protecting against different seizure types in epilepsy patients is shown. Audiogenic seizure‐prone DBA/2 mice are the only model that correctly predicted the antiseizure effects of the various structurally and mechanism‐wise divergent ASMs, including ESM, against different seizure types in epilepsy patients. Thus, in contrast to most other models (including MES and PTZ), the DBA/2 mouse model is not particularly susceptible to certain drug mechanisms, which is similar to the human photosensitivity model. In line with this, we found a highly significant correlation between the antiseizure potencies of ASMs in the DBA/2 mouse model (expressed as median effective dose [ED_50_] in mg/kg) and the average effective oral doses of ASMs in the human photosensitivity model (Figure 2A). A similar correlation was found for the investigational drugs discussed in Section 4 (Figure 2B). It is important to note that, because of the huge differences in body surface area and metabolic rate between rodents and humans, effective doses cannot simply be converted across species, and allometric scaling is needed for dose conversion from animal to human studies.^72^ Such scaling was not applied for the data illustrated in Figure 2A,B, but the significant correlations found for both approved and investigational ASMs indicate that relative drug potencies are similar in both the mouse and human model, which is an important and novel observation.

**TABLE 5:** Antiseizure potencies in animal models of the 13 ASMs that have been tested in the photosensitivity model in epilepsy patients (see Table 1 for details).

Approved antiseizure medications	ED_50_ (mg/kg ip) mouse models						ED_50_ (mg/kg ip) rat models			ED (mg/kg iv) photosensitive baboons (myoclonic seizures)	Effective in photosensitivity model in epilepsy patients	Effective against seizures in epilepsy patients	
	MES (tonic seizures)	sc PTZ (clonic seizures)	6‐Hz (44 mA) focal seizures	Corneal kindled seizures	Focal HPDs in IHK model of mTLE	Clonic audiogenic seizures (DBA/2)	Clonic audiogenic seizures (e.g., GEPRs)	Amygdala kindling model				Focal onset	IGE/DEE
								Focal onset seizures	Secondarily generalized seizures				
Brivaracetam	113	30	4.4	ND	ND	2.4	ND	.68^a^	.68^a^	ND	+	+	+
Carbamazepine	9.8	>50	16.5	144	84	4.4	12.7	15	8	40^b^	+/−	+	+
Cenobamate	9.8	28.5	16.5	ND	ND	14	ND	16.4^c^		ND	+	+	+
Diazepam	5	.29	1.1	.6	1.5	.28	15.8	>10	1.4	1.0	+	+	+
Ethosuximide	>500	136	167^d^	>250	ND	138	1671	>300	>300	>100	+	‐	+
Lamotrigine	5.4	>40	>60	37	>90	3.5	11.1	~2.5		ND	+	+	+
Levetiracetam	>500	>500	>1000	57	580	30	9.0	1.25^a^	1.25^a^	ND	+	+	+
Mephenytoin (methoin)	61	31	ND	ND	ND	ND	ND	ND	ND	ND	+	+	+
Phenobarbital	11.3	13.2	35.3	11	25	3.4	30.14	44	16	15	ND	+	+
Phenytoin	6.7	>50	>60	67	>50	2.5	27.75	50	30	15–50^b^	ND	+	+
Primidone	>40	>40	ND	ND	ND	ND	74.6	>100	>100	>100	+	+	+
Progabide	80	30	ND	ND	ND	50	ND	~400		30–100	+	+	+
Valproate	263	220	310	728	280	43	153	220	190	150–200	+	+	+
Valpromide	56	55	66	ND	ND	ND	ND	~40^c^		150	+	+	+
Vigabatrin	>7000	>7000	ND	308	52	600	<200	<900		360	+	+	+

*Note*: In addition, data for phenobarbital are shown, because phenobarbital is the active metabolite of primidone and is mainly responsible for the antiseizure effect of the latter drug during chronic treatment. Furthermore, phenytoin has been included for comparison. References: Löscher 12, 15, 73, 74; National Institute of Neurological Disorders and Stroke PANAChE database (https://panache.ninds.nih.gov).

Abbreviations: DEE, developmental and epileptic encephalopathy; ED, effective dose; ED_50_, ED in 50% of the animals (as determined by dose‐effect experiments); GEPR, genetic epilepsy‐prone rat; HPD, hippocampal paroxysmal discharge; IGE, idiopathic generalized epilepsy; IHK, intrahippocampal kainate; ip, intraperitoneal; iv, intravenous; MES, maximal electroshock seizure; mTLE, mesial temporal lobe epilepsy; ND, not determined (or not found in the public domain); PTZ, pentylenetetrazole; sc, subcutaneous.

^a^ Minimum active dose.

^b^ Only partially effective.

^c^ Hippocampal kindled rats.

^d^ 32 mA.

Interestingly, such correlation was not found for the audiogenic seizure‐prone rat (Figure 2C), which, at least in part, may be because not all preclinical studies were performed in the same strain of genetically epilepsy‐prone rats (GEPRs; either GEPR‐3 or GEPR‐9). Furthermore, ED_50_s of ASMs determined in mouse and rat models of audiogenic seizures were not correlated (*r* = .4857, *p* = .3556). This indicates that the correlation in drug potencies between DBA/2 mice and photosensitive patients is due to the characteristic feature of these two models to respond to drugs with diverse MOAs, which is not observed in any other animal or human model.

In photosensitive baboons, a positive (albeit not significant) correlation between the antiseizure potencies of ASMs and the average effective oral doses of ASMs in the human photosensitivity model was found (Figure 2D). However, the baboon model did not predict the antiseizure efficacy of ESM and primidone (Table 5). More importantly, photosensitive baboons have produced false positive data concerning drugs that act as competitive antagonists at the NMDA subtype of glutamate receptors, that is, drugs that were associated with a lot of hype and were under development by the pharmaceutical industry in the 1980s/90s (Meldrum, 1986^75^; Porter & Rogawski, 1992^76^; Löscher & Schmidt, 1994^77^), which is discussed in Appendix S1.

This example illustrates the current limitations or gaps in using preclinical models to enhance the translational pipeline for both efficacy and tolerability of investigational ASMs. Thus, as discussed in Appendix S1, rather than relying on a few seizure models as done in the past, a battery of models, both acute and chronic, should be used to characterize a novel compound before moving to clinical trials.^74^, ^78^ Even then, preclinical models could be falsely positive, as illustrated by padsevonil, which was highly effective in a battery of animal models^79^ but failed in large clinical trials (see Section 8). Padsevonil was unfortunately not tested in the photosensitivity model (see also Section 8).

## 6 USE OF THE PHOTOSENSITIVITY MODEL TO MONITOR ADVERSE EFFECTS IN EPILEPSY PATIENTS

As outlined in Appendix S1, as a result of the complex brain alterations associated with epilepsy, epilepsy patients may differ dramatically from healthy volunteers (as used in phase I) in the type and severity of adverse effects. Thus, POP studies with new compounds in the photosensitivity model in epilepsy patients may also disclose adverse effects that were not observed during preclinical development and in phase I tolerability and PK studies in healthy volunteers. One impressive example shown in Table  4 is Org 6370, a rigid amino‐benzobicyclononene derivative. Animal studies with this compound have shown an antiseizure effect against tonic seizures in electrical and chemical mouse and rat models, with a high safety margin.^80^, ^81^ The pharmacological profile of Org 6370 was reminiscent of PHT, but the MOA of Org 6370 was not characterized. In the amygdala kindling model in rats, the drug suppressed focal electrographic seizures (afterdischarges) in the amygdala and cortex but did not reduce motor seizures and in some animals even induced spontaneous seizures at a dose (30 mg/kg ip) that exerted antiseizure effects in acute seizure models in nonepileptic rodents.^80^ In single‐dose and repeated‐dose phase I clinical studies in healthy volunteers, with doses up to 300 mg (single dose) or 200 mg three times daily (t.i.d.), Org 6370 was well tolerated.^80^ No clinically important effects on mood and no drug‐induced sedation were observed. The only dose‐limiting adverse effect was nausea. However, as described in Section 4, unexpected proconvulsant adverse effects occurred in photosensitive epilepsy patients.^37^ A consistent increase in the photosensitivity range was seen following the administration of a single dose of Org 6370, even after adaptation of the original dose of 500 mg to a dose of only 50 mg. Moreover, untoward adverse effects, such as nausea, dizziness, restlessness, drowsiness, and mood disturbances, were reported in all but the patients treated with the lowest dose (50 mg). In the first three patients, the administration of a single dose of Org 6370 (500, 500, and 100 mg, respectively) resulted in a significant increase in the number and severity of evoked and spontaneous myoclonic jerks, and in one patient this was followed by a generalized tonic–clonic seizure. Thus, similar to the example of competitive NMDA receptor antagonists described in Appendix S1, amygdala‐kindled rats correctly predicted the proconvulsant effect of Org 6370 observed in photosensitive epilepsy patients. Based on this finding, further development of Org 6370 was halted.

The taurine derivative taltrimide is another example of unexpected proconvulsant activity in photosensitive epilepsy patients (Table 4). Unlike taurine, an endogenous amino acid that serves a wide variety of functions in the brain,^82^ the lipophilic derivative taltrimide penetrates well into the brain after systemic administration.^83^ Taltrimide was shown to exert antiseizure activity in audiogenic seizure‐prone rats, different chemical seizure models, including PTZ‐induced seizures, and the MES test.^83^, ^84^ Nakagawa and Huxtable^83^ concluded that phthalimidoethanesulfonamides such as taltrimide comprise a novel class of compounds without structural similarity with other ASM types and, as such, may promise new therapeutic entities. However, in a clinical trial of eight patients with photosensitive epilepsy, in whom taltrimide was administered over 6 days (starting with 500 mg for 2 days followed by treatment with 1000 mg twice daily [b.i.d.]), PPR was increased by >50% in four patients, including one who experienced a seizure,^32^ indicating a proconvulsant activity of taltrimide. This was substantiated by a clinical trial in 27 patients with drug‐resistant epilepsy in whom taltrimide was given in gradually increasing doses up to 4.0 g/day.^31^ The frequency of seizures increased significantly during the trial with increasing doses of taltrimide and decreased again in the withdrawal phase of the trial. Of six dropouts, one had status epilepticus, and in two patients increased number or severity of seizures necessitated taltrimide withdrawal.

Apart from these unexpected proconvulsant effects of investigational drugs, the photosensitivity model is commonly used to evaluate whether adverse effects observed in phase I trials in healthy volunteers are also observed in the photosensitive epilepsy patients and whether additional adverse events occur that were not predicted by the phase I data. This is an added advantage of the model that can be used in planning the more laborious and expensive phase IIb trials in patients with drug‐resistant epilepsy.

## 7 USE OF THE PHOTOSENSITIVITY MODEL FOR PK/PHARMACODYNAMIC MODELING

In typical drug trials in the photosensitivity model, drug levels are determined several times after drug administration together with PPR induction (as close in time as possible). Change in photosensitivity range after drug intake in relation to drug plasma levels then allows PK/pharmacodynamic (PD) profiling of the drug under investigation. This is highly valuable information for subsequent larger phase IIa trials in patients with epilepsy.

In addition, because most photosensitive epilepsy patients are under chronic treatment with various ASMs, plasma level analysis of these ASMs and the investigational drug allows determination of drug–drug interactions. Furthermore, a comparison of the investigational drug's effect on PPR in patients with versus without comedication with approved ASMs permits PK/PD modeling that is not possible in subsequent add‐on phase IIb and III trials.

For drugs developed as novel ASMs, BBB penetration and brain target engagement are essential. The use of biomarkers that give accurate information on target engagement, providing confidence that pharmacological activity in the target organ is being achieved, is key in optimizing clinical success.^85^ Apart from in vivo imaging methods (such as positron emission tomography [PET]) used to assess target engagement and receptor occupancy, the photosensitivity EEG response provides excellent evidence for brain target engagement of novel ASMs in humans.

## 8 LIMITATIONS OF THE PHOTOSENSITIVITY MODEL: MYTHS AND FACTS

In our opinion, the main limitation of the photosensitivity model is that it does not allow prediction of the extent of drug resistance to the antiseizure effect of an investigational compound in patients with difficult‐to‐treat types of epilepsy. Unlike drug‐resistant focal epilepsy patients enrolled in classical phase IIa/b trials, photosensitive patients are not necessarily therapy‐resistant, and approximately 25% are ASM‐naïve at the time of the POP trials in the photosensitivity model.^23^ As shown in Table 2 and discussed in Section 2, the percentage of photosensitive patients with no response of the PPR after treatment with approved ASMs ranges from 0% to 83% (median = 0%) and does not reflect the drug resistance with individual ASMs in different types of epilepsy.^24^, ^25^ Thus, photosensitive phase IIb POP trials are a useful tool to qualitatively and quantitatively predict antiseizure effects in subsequent phase IIb add‐on trials in patients with epilepsy but cannot predict whether an investigational compound will differentiate in efficacy and the extent of drug resistance from approved ASMs.

Another potential caveat is that the efficacy of a drug in the photosensitivity model does not allow predicting against which types of epilepsy (other than genetic generalized epilepsies) the drug will be effective. ESM is an example. However, as shown in Table 5, the preclinical profile of ESM clearly indicates that this drug has a narrow spectrum of antiseizure efficacy, with a lack of effects in the MES and kindling models. Thus, this information has to be taken into account together with the effect in the photosensitivity model.

Porter^86^ has proposed that phase IIA data can be more reliably ascertained in a patient population of focal epilepsy, using a novel add‐on trial design for a dose‐finding, safety, and drug interaction study. This study design had been used by Sachdeo et al.^87^ for retigabine (ezogabine) in a relatively small cohort of patients (*N* = 60) with drug‐resistant focal epilepsy before the design and conduct of subsequent pivotal clinical trials. However, this was an open‐label, uncontrolled study without objective efficacy measures and, thus, not a POP study. Franco et al.^88^ also suggested that proof‐of‐concept studies may be conducted by applying the conventional randomized parallel‐group add‐on design using unprovoked seizure counts as the primary end point. In a reanalysis of data from pregabalin add‐on trials, 4 weeks of baseline and 3 weeks of treatment, using a sample size of about 40–50 patients per group, were deemed to be sufficient to demonstrate an antiseizure effect, at least for a drug possessing relatively high efficacy. A similar innovative randomized placebo‐controlled phase IIa study design in patients with drug‐resistant focal epilepsy has more recently been used by Muglia et al.^89^ for the investigational drug padsevonil to maximize signal detection in a small patient population (*N* = 50) for a short duration. At the end of the inpatient period, 30.8% of patients on padsevonil and 11.1% on placebo were ≥ 75% responders (odds ratio = 4.14, *p* < .067). Reduction in median weekly seizure frequency was 53.7% and 12.5% with padsevonil and placebo, respectively (unadjusted *p* < .026), indicating that padsevonil displayed clinically meaningful efficacy in patients with treatment‐resistant epilepsy.^89^ However, subsequent large phase IIb and phase III trials failed to differentiate padsevonil from placebo,^90^ thus questioning the predictivity of small phase IIa add‐on trials as proposed by Porter^86^ and Franco et al.^88^

Apart from using the photosensitivity model for POP studies, transcranial magnetic stimulation (TMS) with EEG/electromyography and responsive neurostimulation (RNS) have been used as biomarker approaches for human testing of ASMs.^91^, ^92^ TMS is widely used for noninvasive and pain‐free assessment of cortical function in brain disorders; cortical responses to TMS pulses likely reflect cortical excitability. The effects of several ASMs, including novel compounds in clinical development, on TMS have been reported, including dose relationships. Of note, certain compounds did not exhibit a dose–response relationship, such as gabapentin and pregabalin, and there are conflicting reports on whether effects on motor threshold can be seen with LEV and valproate. Furthermore, there are substantial differences in TMS protocols between clinical sites and reports in the literature, resulting in inconsistent findings that question the validity of TMS‐EEG as an epilepsy biomarker for drug studies.^91^ RNS involves the implantation of a device that provides a stimulus in response to changes in brain activity that are measured via intracranial electrocorticography. This allows for rapid assessment of an ASM's effects on cortical excitability, in addition to providing continuous EEG data over long periods of time to better understand the durability of the ASM's effect.^92^ However, there is large variability across patients in the changes observed and, as yet, the RNS system has not been used for a POP study with an investigational compound. Thus, concerning POP studies on novel compounds, neither TMS nor RNS is a superior alternative to the photosensitivity model.

Porter^86^, ^93^ also argued that drug effects in the photosensitivity model cannot be used to predict antiseizure effects against focal onset seizures but that the model may produce both false positive and false negative effects, as shown by the PPR‐suppressing effect of ESM^4^ but the lack of such effect by CBZ.^7^ However, as shown here and in the previous review of Yuen and Sims^2^ and based on the finding that PPRs are found in all types of epilepsy, including focal onset and developmental and epileptic encephalopathies (see first part of this review), results of photosensitive POP trials are equally applicable in prediction of drug efficacy in both focal and generalized seizures. In other words, such POP trials do not predict antiseizure efficacy against a specific type of epilepsy; PPR is genetically heterogeneous, so the ability of a drug to suppress the PPR applies to a broader epilepsy spectrum than data gathered in drug‐resistant focal epilepsy patients alone. For instance, in the case of ESM, the data from the photosensitivity model are in line with the efficacy of this drug in idiopathic generalized epilepsies. As discussed in Section 2, the apparent lack of CBZ to suppress PPR in the study of French et al.^7^ is most likely due to the short study duration and the retarded absorption of the ASM, which was not an issue in the positive trial with CBZ in photosensitive patients reported by Binnie et al.^4^

As described in the first part of this review, another often‐raised myth is that a single‐dose experiment with an investigational compound in the photosensitivity model cannot give a prediction of clinical effect and dosing of that new drug, when drugs are normally taken daily over several months during clinical add‐on trials in patients with epilepsy. Reviewing data of 13 ASMs in the photosensitivity model and comparing the efficacious doses in photosensitive POP trials versus the efficacious antiseizure doses, Yuen and Sims^2^ concluded that single‐dose photosensitive POP trials can be a reliable quantitative indicator of investigational ASM efficacy in epilepsy. This is substantiated and extended by the present data, demonstrating a highly significant correlation between effective doses in the model and in the treatment of patients with epilepsy.

Porter^86^ also questioned whether the PPR model may be useful in determining the PK/PD relationship, because such a relationship only applies to photosensitivity, not to response to drugs for focal epilepsy. As outlined by Kasteleijn‐Nolst Trenité^23^ and described in the first part of this review, in the PPR model, PD measurements (PPR ranges) are combined with blood samples at the same time points; this allows a determination of PD/PK relationships per patient per dose with information on time of onset, maximum PPR suppressive effect, and duration of effect related to plasma concentrations of the experimental ASM that can be used to guide subsequent clinical trials.

Based on the lack of a significant change in PPR after single‐dose treatment with CBZ, the value of the PPR in identifying the antiseizure potential of narrow‐spectrum ASMs (such as CBZ) has been questioned.^7^, ^88^ However, the data reviewed here do not support this notion.

One potential caveat of the photosensitivity model is the small number of subjects (usually *n* = 4–6 per dose level) used in most trials in this model. However, typically several dose levels are evaluated in phase IIa POP trials in the PPR model, thus reducing the risk of false positive or negative findings as a result of the small sample size. A recent example is SEL, which was tested at six dose levels in a total of 36 exposures in 27 patients.^50^

There is a general consensus that for a placebo‐controlled phase IIa crossover design with several doses of an investigational compound, 4–6 photosensitive patients are sufficient.^4^, ^7^, ^33^, ^36^, ^37^, ^40^, ^51^, ^58^ This was based on historical data that typically three quarters of photosensitive patients exhibit a PPR response to an ASM (see Section 2). Gurrell et al.^56^ used a statistical approach to define the sample size needed to demonstrate a significant drug effect in the photosensitivity model. The sample size and decision criteria were based on the average least square mean effect over the first 6 h postdose for the standardized photosensitivity range (SPR) in the most sensitive eye condition. The decision criteria were applied to each dose of darigabat. Criterion C1 was at least 95% confident that darigabat effect on reduction in SPR is greater than placebo (this is equivalent to a one‐sided test for statistical significance using an α of .05). Criterion C2 was at least 50% confident that darigabat effect on reduction in SPR is 4 units greater than placebo. The latter criterion assumed an average baseline SPR (across all participants and all treatment periods) of ≥8. To ensure sufficient window to exceed the target value, it was predefined that if the average baseline SPR was <8, the C2 target value was to be adjusted to 50% of the observed average baseline. Based on an assumed conservative within‐participant SD of 2.1 derived from a reanalysis of historical data, eight participants gave 80% probability of meeting criterion C1 for a true reduction in SPR of 2.7 units and 80% probability of meeting both C1 and C2 for a true reduction in SPR of 4.9 units. Based on these calculations, a sample size of approximately eight participants (with the goal of at least six completers) was selected to provide balance in the design and to provide acceptable operating characteristics for prespecified internal decision criteria (Gurrell et al.^56^).

## 9 CONCLUSIONS

Over approximately 50 years of experience with drug testing in the photosensitivity model, a standardized adaptive phase IIa trial protocol has been developed and validated with approved ASMs and has subsequently been used for POP trials with investigational compounds in patients with photosensitive epilepsy. PPR ASM testing offers several advantages as a first evaluation of an investigational drug in epilepsy patients:

1. Drug testing in the photosensitivity model represents a first‐in‐human POP trial with escalating single doses of an investigational drug that shows whether the antiseizure effect seen in animal models can be translated to a PPR‐suppressing effect in epilepsy patients. PPR may provide a nonspecific measure of seizure susceptibility.^88^
2. In contrast to add‐on phase II trials in patients with focal onset epilepsy, drug trials in the photosensitivity model allow comparing the effects of the investigational drug in patients on chronic treatment with ASMs with patients who are not treated by ASMs. This allows for ascertaining whether specific ASMs interfere with the effects of the investigational drugs, for example, by interacting with the same targets. One example are racetams such as LEV, BRV, and SEL, which may compete for the same target (SV2A). For instance, in a POP trial with SEL, patients on LEV showed less marked PPR suppression by SEL than LEV‐naïve patients.^50^
3. Because IPS is performed repeatedly after drug administration, the onset and duration of the PPR‐suppressing effect of an investigational drug can be determined; this may also be used to compare different routes of administration. For instance, in a study in which the PPR‐suppressing effect of LEV and BRV were compared after intravenous administration, BRV acted substantially faster than LEV,^9^ thus substantiating PET studies on the time course of brain target (SV2A) occupancy by these drugs.^94^, ^95^ Such studies are also useful to examine the suitability of a drug formulation for rapid termination of seizure clusters or status epilepticus.
4. The photosensitivity model allows PK/PD modeling after a single drug administration, which is not possible in other types of phase II clinical studies. It also allows for determining the range of effective drug plasma levels of an investigational compound and whether PK drug–drug interactions with approved ASMs occur.
5. Photosensitive POP trials are a useful tool to quantitatively predict efficacy in epilepsy and in aiding dose selection for subsequent larger phase IIb trials with chronic drug administration (see Yuen and Sims^2^).
6. Results of photosensitive POP trials are equally applicable in the prediction of drug efficacy in both focal and generalized seizures. PPR is genetically heterogeneous, and the ability of a drug to suppress the PPR applies to a broader epilepsy spectrum than data gathered in drug‐resistant focal epilepsy patients alone.
7. The photosensitivity model allows determination of whether epilepsy patients exhibit adverse effects after administration of an investigational compound that have not been observed in healthy volunteers (phase I). As discussed in Section 6, Org 6370 and taltrimide are important examples. Epilepsy is a chronic brain disease, and it is thus not surprising that the complex brain alterations associated with epilepsy may affect the adverse effect profile of central nervous system‐active drugs.^78^ In case of unexpected serious adverse effects, an advantage of the single‐blind, adaptive trial design in photosensitive patients is that immediate action can be taken to adapt the dose in the next patients. Information on adverse effects after single doses may be critical to the decision to proceed to further development. Thus, the photosensitivity model reduces the risk of failure or serious adverse effects in larger subsequent trials with chronic drug administration.
8. The PPR phase IIa POP trial model is much less expensive and time‐consuming than open‐label or randomized controlled phase IIa or phase IIb add‐on trials.
9. The risk of false positive or negative findings in the model is low and not higher compared to phase IIa add‐on trials in small cohorts of drug‐resistant epilepsy patients.

As with all types of clinical trials, drug testing in the photosensitivity model is associated with limitations. The model's main limitation is that PPR testing does not predict the extent of resistance of patients' seizures during chronic treatment, nor does it allow differentiating the novel drug in terms of antiseizure efficacy from existing ASMs.

When comparing ASM efficacies in the photosensitivity model with preclinical antiseizure effects, we made a surprising observation in that the audiogenic seizure‐prone DBA/2 mouse model was the only single animal model that correctly predicted the relative potency of the PPR‐suppressing effects of drugs. This observation is important, because it substantiates the notion that results of photosensitive POP trials are equally applicable in the prediction of drug efficacy in both focal and generalized seizures, which is also true for results of drug testing in the DBA/2 mouse model (Bosco et al., 2023)^96^.

It is tempting to speculate that the failure of some novel ASMs (e.g., padsevonil) in large phase III trials in patients with focal onset epilepsy^90^ would have been predicted by an initial POP trial in the human photosensitivity model. Similarly, as shown by the proconvulsant effect of taltrimide and Org 6370 in the photosensitivity model (see Section 6), this model may have predicted the failure of NMDA receptor antagonists in later phase II clinical trials (see Appendix S1).

Overall, the photosensitivity model provides an excellent, unbiased, accurate, inexpensive, and clear POP and PK/PD data‐rich platform for determining the potential efficacy of a novel ASM before entering large add‐on trials with chronic drug administration, irrespective of the type of epilepsy.

## AUTHOR CONTRIBUTIONS

Wolfgang Löscher and Dorothée Kasteleijn‐Nolst Trenité contributed equally to refining the research questions, executing methodological procedures, and writing of the manuscript.

## CONFLICT OF INTEREST STATEMENT

D.K.‐N.T. has in the past 5 years received consultancy fees from UCB, Otsuka, SK, Idorsia, Lundbeck, Jazz, and Praxis. She also receives royalties from Wolters Kluwer (UpToDate). W.L. is cofounder and CSO of PrevEp (Bethesda, MD, USA). He has in the past 5 years received consultancy fees from Lundbeck, Angelini, Clexio, Selene, Axonis, SynapCell, Sintetica, ND Capital, Atlas Venture, Cogent Biosolutions, Ovid, Idorsia, and Addex. We confirm that we have read the Journal's position on issues involved in ethical publication and affirm that this report is consistent with those guidelines.

## Supporting information


Appendix S1.

## Data Availability

No primary data were collected for this study. The original data extraction is available from the corresponding authors upon reasonable request.
