# Supplementary material for: The human photosensitive epilepsy model for clinical proof‐of‐principle trials of novel antiseizure medications: 2. Analysis of drug trials and predictive value of the model
Source: Epilepsia. 2025 May 10;66(8):2619–38. doi: 10.1111/epi.18444 (PMC12371661; doi:10.1111/epi.18444)
Supplement: Supplementary file 1 — Appendix S1. [file EPI-66-2619-s001.pdf]

The human photosensitive epilepsy model for clinical proof-of-principle trials of novel antiseizure medications. 2. Analysis of drug trials and predictive values of the model

Wolfgang Löscher and Dorothée Kasteleijn-Nolst Trenité

Appendix S1

Correlation between antiseizure effects of drugs in animal models and their effects in the human photosensitivity model

With a few exceptions, all currently used antiseizure medications (ASMs) have been developed by animal models of epileptic seizures (Löscher, 2017). Initially, only a few rodent models were used, i.e., the maximal electroshock seizure (MES) test and the s.c. pentylenetetrazole (PTZ) seizure test (Porter and Kupferberg, 2017). However, these tests have several disadvantages (Löscher and Schmidt, 2011). For instance, the MES test is particularly sensitive to drugs that act by modulating sodium channels whereas the PTZ test is sensitive to GABAergic compounds (Löscher, 2016). Furthermore, while the MES test was initially thought to predict antiseizure drug activities against both focal-onset and primary generalized tonic-clonic seizures, this prediction did not hold true for several second and third-generation ASMs, such as levetiracetam (LEV) (Löscher, 2017). Similarly, while the PTZ test was once thought to predict antiseizure activity against absence seizures, the test missed several ASMs, such as lamotrigine (LTG), that act against this type of seizure (Löscher, 2017). Thus, while both the MES and s.c. PTZ tests are still used, they have lost their role as critical gates in ASM development. Instead, they have been largely replaced by models such as the 6-Hz test of focal-onset seizures, different types of kindling models, including amygdala kindling and corneal kindling, and models with spontaneous recurrent

seizures such as the intrahippocampal kainate mouse model of mesial temporal lobe epilepsy (Wilcox et al., 2020).

Another category of animal models uses animals with inherited susceptibility to induction of seizures by sensory stimuli ("reflex seizures"), such as the audiogenic seizure-prone DBA/2 and Frings mouse strains, audiogenic seizure-prone rat strains such as the genetic epilepsy-prone rat (GEPR), i.e., the inbred Sprague-Dawley strains GEPR-3 and GEPR-9, and photosensitive baboons (*Papio papio*)(Löscher and Meldrum, 1984; Löscher, 1984; Faingold et al., 2017). DBA/2 mice have long been used to screen novel ASMs against generalized seizures (Chapman et al., 1984; De Sarro et al., 2017) but their use has decreased because they generally respond to various anticonvulsant drugs and do not discriminate between the efficacy of a new investigational drug possesses compared to clinically established ASMs, particularly in difficult-to-treat types of epilepsy (Löscher, 2016). On the other hand, DBA/2 mice identified the antiseizure efficacy of LEV (Gower et al. 1992), while LEV was ineffective in the MES and s.c. PTZ tests (Löscher and Hönack, 1993) and thus failed in the Anticonvulsant Screening Program (ASP) of the NINDS in the early 1990s (Löscher et al., 2016). The latter fact was one of the reasons for adding several animal models of seizures and epilepsy to the ASP (and later ETSP) programs of the NINDS (Wilcox et al., 2013; Wilcox et al., 2020). The DBA/2 mouse model was also decisive in identifying the antiseizure activity of brivaracetam and seletracetam (Klitgaard et al., 2016).

The complex mechanisms underlying audiogenic seizure susceptibility in DBA/2 mice have been studied extensively (Faingold et al., 2017). In addition to audiogenic seizures, these mice also display a genetically based increased susceptibility to absence seizures and handling-induced seizures. DBA/2 mice exhibit a polygenic inheritance pattern, which affects their susceptibility to audiogenic seizures. Specific genetic loci have been identified that correlate with this condition, including *Asp1*, *Asp2*, and *Asp3*, located on chromosomes 12, 4, and 7 (Bosco et al., 2023). These loci are involved in the regulation of Ca^{2+}-ATPase

activity, which is important for synaptic function and neurotransmitter release from synaptic vesicles. DBA/2 mice also express an astrocyte-specific Kcnj10 deletion that has been shown to disrupt the activity of inward rectifying potassium (Kir) 4.1 channels and uptake glutamate; this results in low seizure threshold of audiogenic mice compared with the seizure-insensitive C57BL/6J mice (Bosco et al., 2023). Furthermore, a single nucleotide polymorphism (C1473G) has been identified in the central nervous system affecting the specific 5-HT synthesizing enzyme, tryptophan hydroxylase, resulting in a significant reduction in 5-HT synthesis in DBA/2 mice (Zhang et al., 2004). Indeed, there is considerable evidence supporting a deficit in serotonergic neurotransmission and alteration in serotonergic receptors in DBA/2 mice (Faingold et al., 2017). Furthermore, imbalances in excitatory glutamatergic and inhibitory GABAergic neurotransmission are implicated in audiogenic seizure mechanisms in this mouse strain (De Sarro et al., 2017). Additionally, disturbances in adenosine neurotransmission have been observed, further complicating the seizure mechanisms (Bosco et al., 2023). Neuroimaging in DBA/2 mice found increases in c-fos expression following audiogenic seizures primarily in subcortical brainstem auditory nuclei (Le Gal La Salle and Naquet, 1990). In addition to using DBA/2 mice as a model of seizures, these mice are increasingly been used as a model of SUDEP (sudden unexpected death in epilepsy) (Bosco et al., 2023).

As described in section 5, audiogenic seizure-prone DBA/2 mice are the only model that correctly predicted the antiseizure effects of the various structurally and mechanism-wise divergent ASMs, including ESM, against different seizure types in epilepsy patients. In line with this, we found a highly significant correlation between the antiseizure potencies of ASMs in the DBA/2 mouse model (expressed as ED_{50} in mg/kg) and the average effective oral doses of ASMs in the human photosensitivity model (Fig. 2A). A similar correlation was found for the investigational drugs discussed in section 8 (Fig. 2B). Interestingly, in contrast to DBA/2 mice, such correlation was not found for the audiogenic seizure-prone rat (Fig. 2C).

This is also true for the photosensitive baboon model that did not predict the antiseizure efficacy of ESM and primidone (Table 5). More importantly, photosensitive baboons have produced false positive data concerning drugs that act as competitive antagonists at the NMDA subtype of glutamate receptors, i.e., drugs that were associated with a lot of hype and were under development by the pharma industry in the 1980s/90s (Meldrum, 1986; Porter and Rogawski, 1992; Löscher and Schmidt, 1994). Based on experiments with competitive NMDA receptor antagonists (CPG 37849 and CGP 39551) in photosensitive Senegalese baboons, Chapman et al. (1991) concluded that the high antiseizure potency of these compounds after oral administration enhances the possibility of clinical evaluation of this class of compound. However, in contrast to the findings in baboons, CPG 37849 and CGP 39551 exerted only weak antiseizure effects in fully amygdala-kindled rats and did not increase the focal seizure threshold, indicating a lack of effect against focal-onset seizures (Löscher and Hönack, 1991a). Furthermore, while no side effects were evident in the baboons at any of the doses tested p.o., using single or repeated administration (Chapman et al., 1991), the weak anticonvulsant effects of the NMDA receptor antagonists in kindled rats were associated with profound untoward behavioral effects, including hyperactivity and stereotypies that were not observed in non-kindled rats (Löscher and Hönack, 1991a). We concluded that epileptogenic brain alterations (as initiated by kindling) alter the responses to competitive NMDA receptor antagonists and that the findings in kindled rats might indicate that such drugs may also produce psychotomimetic effects in humans with focal-onset epilepsy (Löscher and Hönack, 1991a,b). Indeed, the first clinical trial with a competitive NMDA receptor antagonist (D-CPP-ene) in patients with focal-onset epilepsy induced severe psychosis-like adverse effects in all patients, requiring hospitalization in most patients and premature termination of the trial, while the drug had been well tolerated by healthy volunteers (Sveinbjornsdottir at al., 1993). Seizure control was worsened in three patients and unchanged in the others; the patient with the highest plasma concentration of D-CPP-ene

developed a complex focal status epilepticus. Given these disappointing clinical data, the strategy of developing competitive NMDA receptor antagonists for epilepsy was discontinued, although novel agents that target different NMDA receptor subtypes may offer new avenues for capturing the therapeutic potential of targeting this important receptor class (Egunlusi and Joubert, 2024; Hanson et al., 2024). This example underlines the usefulness of the amygdala kindling model for avoiding false positive data. This model was also the first that predicted the high efficacy of LEV in focal-onset epilepsies (Löscher and Hönack, 1993).

We do not argue that the DBA/2 mouse could replace other seizure models used in the discovery and differentiation phases of preclinical ASM development. However, as shown here, data from the DBA/2 model can be used to predict potency and efficacy in POP trials in photosensitive patients and thus de-risk clinical development. However, DBA/2 mice did not predict the failure of competitive NMDA receptor antagonists as novel epilepsy therapies but CPG 37849 and CGP 39551 were quite potent in suppressing clonic audiogenic seizures at doses below those inducing adverse effects (Chapman et al., 1991). This is one of many examples that ASM development should not rely on a single model.

References

Bosco,F., Guarnieri,L., Leo,A., Tallarico,M., Gallelli,L., Rania,V., Citraro,R., and De Sarro,G. (2023). Audiogenic epileptic DBA/2 mice strain as a model of genetic reflex seizures and SUDEP. Front Neurol. *14*, 1223074.

Chapman,A.G., Croucher,M.J., and Meldrum,B.S. (1984). Evaluation of anticonvulsant drugs in DBA/2 mice with sound-induced seizures. Arzneim. -Forsch. (Drug Res.) *34*, 1261-1270.

Chapman,A.G., Graham,J.L., Patel,S., and Meldrum,B.S. (1991). Anticonvulsant activity of two orally active competitive N-methyl-D-aspartate antagonists, CGP 37849 and CGP 39551, against sound-induced seizures in DBA/2 mice and photically induced myoclonus in *Papio papio*. Epilepsia *32*, 578-587.

De Sarro,G., Russo,E., Citraro,R., and Meldrum,B.S. (2017). Genetically epilepsy-prone rats (GEPRs) and DBA/2 mice: Two animal models of audiogenic reflex epilepsy for the evaluation of new generation AEDs. Epilepsy Behav. *71*, 165-173.

Egunlusi,A.O. and Joubert,J. (2024). NMDA Receptor Antagonists: Emerging Insights into Molecular Mechanisms and Clinical Applications in Neurological Disorders. Pharmaceuticals. (Basel) *17*, 639.

Faingold,C., Tupal,S., and N'Gouemo,P. (2017). Genetic Models of Reflex Epilepsy and SUDEP in Rats and Mice. In Models of seizures and epilepsy. 2nd edition., A.Pitkänen, P.S.Buckmaster, A.S.Galanopoulou, and S.L.Moshé, eds. (London: Academic Press), pp. 441-453.

Gower,A.J., Noyer,M., Verloes,R., Gobert,J., and Wülfert,E. (1992). ucb L059, a novel anti-convulsant drug: pharmacological profile in animals. Eur. J. Pharmacol. *222*, 193-203.

Hanson,J.E., Yuan,H., Perszyk,R.E., Banke,T.G., Xing,H., Tsai,M.C., Menniti,F.S., and Traynelis,S.F. (2024). Therapeutic potential of N-methyl-D-aspartate receptor modulators in psychiatry. Neuropsychopharmacology *49*, 51-66.

Klitgaard,H., Matagne,A., Nicolas,J.M., Gillard,M., Lamberty,Y., De Ryck,M., Kaminski,R.M., Leclercq,K., Niespodziany,I., Wolff,C., Wood,M., Hannestad,J., Kervyn,S., and Kenda,B. (2016). Brivaracetam: Rationale for discovery and preclinical profile of a selective SV2A ligand for epilepsy treatment. Epilepsia *57*, 538-548.

Le Gal,L.S. and Naquet,R. (1990). Audiogenic seizures evoked in DBA/2 mice induce c-fos oncogene expression into subcortical auditory nuclei. Brain Res. *518*, 308-312.

Löscher,W. and Meldrum,B.S. (1984). Evaluation of anticonvulsant drugs in genetic animal models of epilepsy. Fed. Proc. *43*, 276-284.

Löscher,W. and Hönack,D. (1991). Anticonvulsant and behavioral effects of two novel competitive N- methyl-D-aspartic acid receptor antagonists, CGP 37849 and CGP 39551, in the kindling model of epilepsy. Comparison with MK-801 and carbamazepine. J. Pharmacol. Exp. Ther. *256*, 432-440.

Löscher,W. and Hönack,D. (1991). Responses to NMDA receptor antagonists altered by epileptogenesis. Trends Pharmacol. Sci. *12*, 52.

Löscher,W. and Hönack,D. (1993). Profile of ucb L059, a novel anticonvulsant drug, in models of partial and generalized epilepsy in mice and rats. Eur. J. Pharmacol. *232*, 147-158.

Löscher,W. and Schmidt,D. (1994). Strategies in antiepileptic drug development: is rational drug design superior to random screening and structural variation? Epilepsy Res. *17*, 95-134.

Löscher,W. and Schmidt,D. (2011). Modern antiepileptic drug development has failed to deliver: ways out of the current dilemma. Epilepsia *52*, 657-678.

Löscher,W., Gillard,M., Sands,Z.A., Kaminski,R.M., and Klitgaard,H. (2016). Synaptic Vesicle Glycoprotein 2A Ligands in the Treatment of Epilepsy and Beyond. CNS Drugs *30*, 1055-1077.

Löscher,W. (2016). Fit for purpose application of currently existing animal models in the discovery of novel epilepsy therapies. Epilepsy Res. *126*, 157-184.

Meldrum,B.S. (1986). Drugs acting on amino acid neurotransmitters. Adv. Neurol. *43*, 687-706.

Porter,R.J. and Rogawski,M.A. (1992). New antiepileptic drugs: from serendipity to rational discovery. Epilepsia *33 Suppl 1*, S1-S6.

Sveinbjornsdottir,S., Sander,J.W.A.S., Upton,D., Thompson,P.J., Patsalos,P.N., Hirt,D., Emre,M., Lowe,D., and Duncan,J.S. (1993). The excitatory amino acid antagonist D-CPP-ene (SDZ EAA-494) in patients with epilepsy. Epilepsy Res. *16*, 165-174.

Wilcox,K.S., Dixon-Salazar,T., Sills,G.J., Ben Menachem,E., White,H.S., Porter,R.J., Dichter,M.A., Moshe,S.L., Noebels,J.L., Privitera,M.D., and Rogawski,M.A. (2013). Issues related to development of new antiseizure treatments. Epilepsia *54 Suppl 4*, 24-34.

Wilcox,K.S., West,P.J., and Metcalf,C.S. (2020). The Current Approach of the Epilepsy Therapy Screening Program Contract Site for Identifying Improved Therapies for the Treatment of Pharmacoresistant Seizures in Epilepsy. Neuropharmacology *166*, 107811.

Zhang,X., Beaulieu,J.M., Sotnikova,T.D., Gainetdinov,R.R., and Caron,M.G. (2004). Tryptophan hydroxylase-2 controls brain serotonin synthesis. Science *305*, 217.
